# We must not fool ourselves: A reply to Sethi et al. on the use of BirdNET to classify neotropical birdcalls

**DOI:** 10.1073/pnas.2419635121

**Published:** 2024-12-11

**Authors:** Carlos B. de Araújo

**Affiliations:** ^a^Atlantic Forest Biodiversity Observatory, Instituto de Biología Subtropical, Universidad Nacional de Misiones & Consejo Nacional de Investigaciones Científicas y Técnicas, Puerto Iguazú 3370, Argentina

The acousticmonitoring (PAM) of biodiversity has gained traction in recent years, even though classifying species within a recording could be challenging in places where acoustic diversity is high. Among the classification algorithms recently developed, BirdNET is probably the most promising. BirdNET was built to recognize over six thousand bird species and was trained using data from Xeno-canto and the Macaulay Library. Despite its huge potential, BirdNET is known to struggle with noisier recordings (1), reducing its accuracy for PAM.

In this context, Sethi et al. (2) recently evaluated BirdNET’s performance across four regions—Norway, Taiwan, Costa Rica, and Brazil—processing approximately 152,000 h of sound recordings. They limited their analysis to confidence scores higher than 0.8, yielding the detection of 379 species. Limiting the analysis to the species with at least 50 detections provided 16 species for Brazil and 19 for Costa Rica. The authors found BirdNET can deliver “reliable monitoring for many bird species across four large and diverse datasets,” while acknowledging limitations in Costa Rica and Brazil, where bird species remain underrepresented in global acoustic libraries.

While the paper has merit, I believe some issues reduce its value. First, the authors describe the study site in Brazil as “tropical forest in the Amazon,” yet 12 of the 16 species detected by BirdNET are typical open-area species (3). Supplementary material reveals the sites were actually in riparian forests and pastures. In perspective, a single recorder in Pará’s tropical forests should detect around 56 species in the first day [144 one-min samples; (4)], while 25 recorders (as in the study) would detect 245 species. Caatinga is among the poorest of Brazilian Biomes, and yet 107 species should be detected using 25 recorders in the first day (3,600 min). In contrast, BirdNET found enough data for 16 species in 46,000 one-min samples in Brazil, and 19 species in Costa Rica, from approximately 1.5 million one-min samples.

The detection of species like *Pitangus sulfuratus, Tyrannus melancholicus*, *Crotophaga ani, Vanellus chilensis, Volatina jacarina,* and *Elaenia flavogaster* in the tropics highlight BirdNET’s ability in identifying common species, that were probably trained with lots of data. It also highlights BirdNET’s inefficiency with “not so common” species. The author suggests that Brazil remains underrepresented in global libraries of avian vocalizations, and more data would be necessary to train the models. Nevertheless, they overlook the fact that together, WikiAves and Fonoteca Neotropical Jacques Vielliard hold nearly 360,000 sound recordings of Brazilian birds (5).

Richard Feynman, the renowned physicist, once said, “The first principle is that you must not fool yourself—and you are the easiest person to fool.” While BirdNET holds promise, its current limitations in tropical regions must be fully addressed before it can be applied at scale. In that sense, the study of Sethi et al. would have made a better contribution by focusing on the limitations of BirdNET in the Neotropics and specially by offering solutions to improve its performance.
